# Editorial: Evolution of phytochemicals and phytotherapies in the treatment and management of cancer: targeted strategies in cancer precision medicine

**DOI:** 10.3389/fphar.2023.1326474

**Published:** 2023-11-02

**Authors:** Mohd Adnan, Visweswara Rao Pasupuleti, Mitesh Patel

**Affiliations:** ^1^ Department of Biology, College of Science, University of Ha’il, Ha’il, Saudi Arabia; ^2^ Center for International Collaboration and Research, REVA University, Bangalore, Karnataka, India; ^3^ Research and Development Cell, Department of Biotechnology, Parul Institute of Applied Sciences, Parul University, Vadodara, India

**Keywords:** phytomedicine, precision medicine, natural products, phytotherapies, cancer

There is a rising recurrence of cancers in humans and related complications of chemotherapeutic agents that reduce the clinical potency of numerous anticancer agents, including those which are currently in use. Hence, there is a consistent need for the development of alternative or synergistic anticancer drugs with lower side complications. Plants are predicted to have innumerable functional phytochemical constituents with potent features, and aid as prime source and candidates for the discovery of anticancer drugs. Therefore, considering the fact, this Research Topic of Frontiers in Pharmacology aims to cover the current trends and advancements in phytotherapies with an emphasis on targeted strategies in cancer precision medicine. Total of 17 manuscripts, which includes 10 research and 7 review articles were published in this Research Topic covering the recent advances in phytochemicals and phytotherapies for targeted cancer therapy.

First paper focuses on the treatment of rhabdomyosarcoma (RMS), the most common type of pediatric soft tissue sarcoma. Since RMS efficiently activates mechanisms of resistance to therapies, despite improvements, the prognosis remains still largely unsatisfactory. Here, authors have showed the therapeutic properties of PBI-05204, an extract from *Nerium oleander* containing the CG oleandrin, which is already studied in phase I and II clinical trials for cancer patients, were investigated, *in vitro* and *in vivo*, against FN- and FP-RMS cancer models. They found that PBI-05204 efficiently counteracted the transformed and intrinsically radioresistant phenotype of RMS by concomitantly inducing cytostatic and cytotoxic effects, promoting RT-induced G2 cell cycle arrest and restraining the ability of RMS cells to repair RT-induced DNA damage. These results suggest that PBI-05204 could have therapeutic and radiosensitizing properties on RMS (Vaccaro et al.).

Second paper highlights the role of dietary flavone apigenin (4′,5,7-trihydroxyflavone) in interacting and inhibiting the topoisomerase 1 and upregulating the CD26/DPP4 on colorectal carcinoma cells (CRC). They observed a unique synergistic interaction with the CRC chemotherapeutic agent irinotecan. The interplay between apigenin and irinotecan was not observed when apigenin was combined with other chemotherapeutic drugs including the topoisomerase 2 inhibitors doxorubicin or etoposide. Authors conclude that apigenin has a unique fit into the Topo1-DNA functional complex that leads to direct inhibition of Topo1 activity, and suggest that this is the basis for the exceptional interaction with the CRC drug irinotecan. A combined action of these two agents may therefore exert a role to limit local signals that facilitate tumour progression (Fux et al.).

Third paper focuses on locating the potential interactions of the diosgenin-regulated proteins and attributable pathways implicated in breast cancer using a range of system biology techniques, *i.e.,* gene ontology (GO) analysis, molecular docking, and molecular dynamics (MD) simulations, and reinforcing its findings using diverse functional biomarkers using *in vitro* experiments. Authors identified the probable action of the diosgenin against breast cancer via FoxO, PI3K-Akt, p53, Ras, and MAPK signaling pathways. This work will open the way for the development of novel therapeutic techniques and/or medication candidates for breast cancer (Khanal et al.).

Fourth paper presents a comprehensive review focussed on the potential anticancer benefits of bromelain, analyzing the cytotoxic, apoptotic, necrotic, autophagic, immunomodulating, and anti-inflammatory effects in cancer cells and animal models. Bromelain, the main medicinal component of pineapple, is an enzyme with numerous pharmacological properties, as it can act on different health disorders, including osteoporosis and osteoarthritis, diarrhea, chronic wounds, surgical debridement, edema, inflammation, and cancer. The anticancer properties of bromelain are extensively documented *in vitro* experiments, but such demonstrations *in vivo* animal models are far less. Here, authors have presented the novel approaches to cancer chemotherapy, which are warmly urgent and bromelain could be regarded as an important tool in the cancer fight (Pezzani et al.).

Fifth paper aimed to investigate the effects and mechanism of HQ (*Astragalus membranaceus* (Fisch.) Bunge (Huang Qi in Chinese), a well-known Chinese herbal medicine, and its bioactive ingredients FMNT (isoflavonoid, formononetin) and CS (isoflavonoid, calycosin) against colon cancer using network pharmacology analysis coupled with experimental validation and molecular docking. The findings suggested that the HQ exerted good therapeutic effects against colon cancer by mainly inhibiting the ERK1/2 signaling pathway. FMNT and CS were two bioactive ingredients responsible for the inhibitory effects of HQ against colon cancer. The current study expands our knowledge pertaining to the effects and mechanism of HQ against colon cancer, and suggests that FMNT and CS will hopefully serve as prospective compounds for colon cancer treatment (Hu et al.).

Sixth paper presents a systematic review focussed on the wide range of anticancer effects of licochalcones in gastric, lung, colon, breast, liver, and bladder cancer. After analyzing and collating the literature, authors concluded that the regulation of multiple signaling pathways by licochalcones includes the EGFR/ERK, PI3K/Akt/mTOR, p38/JNK, JAK2/STAT3, MEK/ERK, Wnt/β-catenin, and MKK4/JNK signaling pathways, which is the key to their antineoplastic effects. Among all the examined licochalcones, licochalcone A (LA) not only has antineoplastic effects, but also can be used to reduce drug efflux from cancer cells and reduce adverse reactions caused by other antitumor drugs. Authors therefore believe that the use of LA as an adjunct to anticancer drugs holds great promise (Deng et al.).

Seventh paper summarizes the antitumor activity and associated mechanisms of the organosulfur compounds of garlic in breast carcinoma. Authors presented in this review that garlic extract, its bioactive compounds, and their use in nanoformulations can prevent breast cancer in all of its stages, including initiation, promotion, and progression. Additionally, these bioactive compounds may affect cell signaling for cell cycle arrest and survival along with lipid peroxidation, nitric oxide synthase activity, epidermal growth factor receptor, nuclear factor kappa B (NF-κB), and protein kinase C in breast carcinoma. Altogether, in this review, authors have demonstrated the anticancer potential of garlic phytoconstituents and its nanoformulations as beneficial nutraceuticals and pharmaceuticals for the efficient management of human breast cancer (Pandey et al).

Eighth paper discusses the specific mechanisms and molecular targets of calotropin, a pharmacologically active compound isolated from milkweed plants like *Calotropis procera, Calotropis gigantea,* and *Asclepias currasavica* in cancer treatment. Calotropin is identified as a highly potent cardenolide that has a similar chemical structure to cardiac glycosides (such as digoxin and digitoxin). During the last few years, cytotoxic and antitumor effects of cardenolides glycosides have been reported more frequently. Among cardenolides, calotropin is identified as the most promising agent. Authors demonstrated that calotropin can be a potential chemotherapeutic/chemopreventive adjunctive agent in cancer pharmacotherapeutic management (Rajkovic et al.).

Ninth paper demonstrates the antitumor effects and potential mechanisms underlying the impact of 5-Demethylnobiletin (5-DMN), the active ingredient in citrus polymethoxyflavones on Glioblastoma (GBM) for the first time both *in vitro* and *in vivo*. Authors elucidated that 5-DMN promoted G0/G1 phase arrest and apoptosis in glioblastoma cells by restraining the ERK1/2, AKT and STAT3 signaling pathways. The results will help to evaluate the potential applications of 5-DMN as a clinical agent for glioblastoma (Zhang et al.).

Tenth paper investigates the inhibitory mechanisms of Polymethoxyflavonoids (PMFs) from *Citrus reticulata* ‘Chachi’ (CRCP) on nasopharyngeal carcinoma (NPC) growth *in vivo* and *in vitro*. The results of this study demonstrated for the first time that heptamethoxyflavone (HMF) purified from CRCP significantly inhibited the proliferation and induced apoptosis of NPC cells (CNE-2 and 5–8F), and also inhibited NPC cell migration and invasion. The results of the tumor transplantation experiment in nude mice confirmed the inhibitory effect of HMF on NPC cell growth *in vivo*. These findings provide a preliminary experimental basis for treating NPC and the development and utilization of PMFs in CRCP (Yang et al.).

Eleventh paper aimed to determine whether the administration of cocoa bean extract reduces doxorubicin-induced organ damage in mice with Ehrlich ascites carcinoma (EAC) without compromising doxorubicin efficacy. Authors demonstrated the protective effect of cocoa extract (COE) against doxorubicin-induced organ toxicities (heart, liver, and kidney), but also indicated synergistic potential with the anticancer activity of doxorubicin. Furthermore, the study also demonstrated the efficacy of COE to neutralize the free radicals generated by doxorubicin; maintaining the cell integrity, along with the inherent anti-cancer properties; and prolonging the survival time of EAC mice. These findings exhibit the promising nutraceutical properties toward cardioprotective, hepatoprotective, and nephroprotective effects, when supplemented with doxorubicin (Patil et al.).

Twelfth paper aims to summarize and understand the mechanisms behind the anticancer potential of sulforaphane (SFN). Authors from this review concludes that SFN provides cancer protection through the alteration of various epigenetic and non-epigenetic pathways. It is a potent anticancer phytochemical that is safe to consume with minimal side effects. However, there is still a need for further research regarding SFN and the development of a standard dose. This will point to new therapeutic perspectives towards the possible development of new sulforaphane-based anticancer drugs (Ali et al.).

Thirteenth paper focuses on exploring the anticancer attributes of Cucurbitacin-B (Cur-B) against androgen-dependent prostate adenocarcinoma (PCa) LNCaP cells. Authors concluded that Cur-B suppressed the growth of PCa cells, which is associated with apoptosis induction, caspase activation, ROS generation, dissipation of mitochondrial membrane potential, and modulation of genes associated with apoptosis and cell cycle arrest. As a result, the data suggests the plausible potential of Cur-B as an alternative and complementary medicine for the treatment of PCa (Alafnan et al.).

Fourteenth paper focuses on details regarding the traits of the pentacyclic triterpenes known as boswellic acids (BAs), their roles as anti-cancer agents, the mechanism underlying their activities, and the function of their semi-synthetic derivatives in managing and treating certain cancers. The review also explores the biological sources of BAs, how they are conserved, and how biotechnology might help preserve and improve *in vitro* BA production. Authors conclude that the BAs and their semi-synthetic derivatives are effective against a broad spectrum of cancer cell lines. This comprehensive review can be helpful for researchers to gain more information about BAs and BA-based medications for efficient and cost-effective cancer treatments (Trivedi et al.).

Fifteenth paper is the first study to summarize the research progress on various compounds, including natural products and derivatives, that target the canonical Wnt pathway in lung cancer (LC) to develop safer and more targeted drugs or alternatives. Authors have particularly presented the impact of Wnt in mediating clinically relevant therapies for LC, such as chemotherapy, radiotherapy, EGFR-TKI, immunotherapy, and even anti-angiogenic therapy. Therefore, Wnt combination therapy is a good strategy for overcoming drug resistance in LC, and should be an overriding direction for future research. Many naturally occurring small molecules used as Wnt inhibitors have good effects, when used in combination with chemotherapy and targeted drugs, with the advantage of inhibiting proliferation and overcoming drug resistance (Shen et al.).

Sixteenth paper aimed to explore the efficacy of diallyl trisulfide (DATS) combined with cisplatin (DDP) for gastric cancer treatment and its underlying mechanism based on network pharmacology. Here, authors demonstrated that the combination of DDP and DATS significantly increased cytotoxicity and cell apoptosis compared to the DATS or DDP treatment group *in vitro*. In addition, continuous intraperitoneal injection of DATS markedly improved the tumor inhibitory effect of DDP in the SGC-7901 tumor-bearing mouse model. Authors further revealed that the combination of DATS and DDP synergistically enhanced antitumor activity by regulating endoplasmic reticulum stress and inhibiting STAT3/PKC-δ and MAPK signaling pathways. This study offers a new adjuvant strategy based on DATS and DDP for the treatment of gastric cancer (Lv et al.).

Seventeenth and final paper report the synthesis of methotrexate-conjugated zinc oxide nanoparticles (MTX-ZnONPs) and their high efficacy against lung cancer cells. The results of the current study have illustrated the conjugation of MTX with ZnONPs with high drug-loading efficiency. Authors emphasized the efficient delivery of MTX to lung cancer cells via ZnONPs as nanocarriers and showed that MTX could induce apoptosis in lung cancer cells via both caspase-dependent and caspase-independent pathways (Mishra et al.).

Existing therapeutic approaches to treat cancer in humans are invasive and often exhibit long-lasting side effects. Furthermore, there are a limited number of treatments available to treat different types of cancers, which represents a major challenge for cancer drug discovery. It is therefore necessary to develop new anticancer drugs. Improving fundamental understanding of the mechanisms underlying the cascade of cancer progression is of key importance towards transforming the landscape of cancer research and developing new and improved treatments of numerous cancerous cell types. Finally, the Guest Editors would like to sincerely thank all the authors and reviewers for their valuable contributions.

